# Involvement of miR-20a in Promoting Gastric Cancer Progression by Targeting Early Growth Response 2 (EGR2)

**DOI:** 10.3390/ijms140816226

**Published:** 2013-08-06

**Authors:** Xiangsheng Li, Zhichao Zhang, Ming Yu, Liqi Li, Guangsheng Du, Weidong Xiao, Hua Yang

**Affiliations:** Department of General Surgery, Xinqiao Hospital, Third Military Medical University, Xinqiao Road, Chongqing 400037, China; E-Mails: xiangsonlee@163.com (X.L.); zhichao_zhang@163.com (Z.Z.); yumimianbao@163.com (M.Y.); liliqi198610@163.com (L.L.); guangsheng_du@126.com (G.D.)

**Keywords:** gastric cancer, miR-20a, early growth response 2, carcinogenesis, chemoresistance

## Abstract

Gastric cancer (GC) is one of the most common cancers, with high incidences in East Asia. microRNAs (miRNAs) play essential roles in the carcinogenesis of GC. miR-20a was elevated in GC, while the potential function of miR-20a was poorly understood. miR-20a expression was examined in GC tissues and cell lines. The effects of miR-20a on the growth, migration, invasion, and chemoresistance of GC cells were examined. Luciferase reporter assay and Western blot were used to screen the target of miR-20a. miR-20a was increased in GC tissues and cell lines. miR-20a promoted the growth, migration and invasion of GC cells, enhanced the chemoresistance of GC cells to cisplatin and docetaxel. Luciferase activity and Western blot confirmed that miR-20a negatively regulated EGR2 expression. Overexpression of EGR2 significantly attenuated the oncogenic effect of miR-20a. miR-20a was involved in the carcinogenesis of GC through modulation of the EGR2 signaling pathway.

## 1. Introduction

Gastric cancer (GC) is one of the most common cancers and the second most common malignancy of cancer death worldwide, especially in East Asia [1]. Despite a steady decrease in GC incidence and mortality during the last decade, GC still ranked second in global cancer mortality. The carcinogenesis of GC was complicated involving dysregualtion of oncogenes and tumor suppressors [2]. Recently, emerging evidence found that a new group of RNAs, known as microRNAs (miRNAs), regulated a large variety of genes, including both oncogenes and tumor suppressor genes [3].

miRNAs were a family of endogenous, non-coding small RNAs (approximately 20–25 nucleotides), which negatively regulated gene expression by inhibiting translation or inducing mRNA degradation via binding to the 3′ untranslated region (3′ UTR) of target mRNAs. miRNAs played critical roles in the development, proliferation, differentiation, metabolism, and apoptosis. In addition, aberrant expression of miRNAs was related to carcinogenesis [4]. Abnormal expression profiles of miRNAs had been reported in numerous cancers, including breast, colon, lung, prostate, and GC [5–8]. miRNAs functioned as oncogenes or tumor suppressors by regulating different target gene expression in different cancers. miR-9, miR-22, and miR-146a had all been shown to act as tumor suppressors [9–11], whereas miR-19, miR-23a, and miR-301a had been shown to function as oncogenes [12–14]. These studies suggested that dysregulation of miRNAs might be involved in carcinogenesis and cancer progression.

miR-20a belonged to the miR-17-92 cluster, which were widely overexpressed oncogenes in diverse cancer subtypes [14]. Previous studies had shown that certain cancer suppressors, such as BH3-only protein (BIM), Phosphatase and tensin homolog (PTEN), were direct targets of the miR-17-92 cluster [12,15]. In human cervical cancer cells, miR-20a was reported to promote cancer cell migration and invasion by targeting Tankyrase 2 (TNKS2) [16]. Similarly, in osteosarcoma, miR-20a increased the metastatic potential of osteosarcoma cells by targeting Fas, indicating an oncogenic function of miR-20a [17]. However, the role of miR-20a in the progression of GC and its underlying mechanism remained unclear.

In this study, we explored the biological effects and the potential mechanisms of miR-20a in GC by detecting the expression of miR-20a in GC, validating previous finding that miR-20a was elevated in GC. Overexpression of miR-20a promoted the growth, migration, invasion, as well as chemoresistance of GC cells. By bioinformatics analysis, we predicted the tumor-suppressor, early growth response 2 (EGR2), as a putative target of miR-20a. Subsequent luciferase activity assay and Western blot confirmed that miR-20a repressed the expression of EGR2 by inducing EGR2 mRNA decay. Overexpression of EGR2 significantly attenuated the oncogenic effect of miR-20a.

## 2. Results

### 2.1. miR-20a Was Increased in GC Tissue Samples and Cell Lines

**Figure 1:**
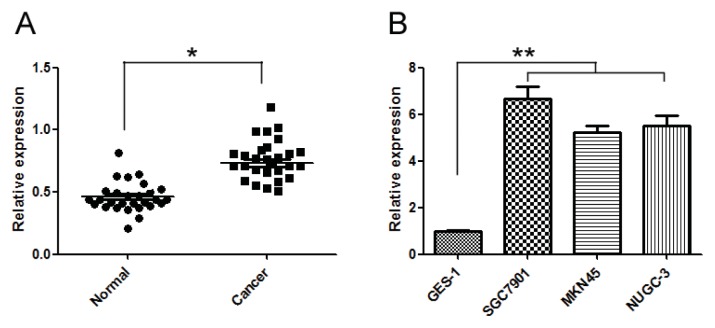
miR-20a was increased in gastric cancer (GC) tissue samples and cell lines. (**A**) qRT-PCR for miR-20a was performed using 28 GC tissue samples and matched with adjacent non-tumour normal tissues; (**B**) qRT-PCR for miR-20a was performed using 3 GC cell lines and human gastric mucosa cell line GES-1. The data represented triplicate measurements. *p < 0.05, **p < 0.01 compared with control.

To validate the expression levels of miR-20a, we conducted quantitative real time PCR (qRT-PCR) in 28 GC tissues and the corresponding normal tissues and 3 GC cell lines and a gastric epithelial cell line. Expression of miR-20a was significantly increased in GC tissues and cell lines (Figure 1A,B). The clinicopathological features of 28 patients with GC were shown in Table 1, and miR-20a was correlated with the metastasis of GC patients, while the miR-20a expression had no relationship with other clinicopathological parameters. Thus, it was suggested that elevation of miR-20a in GC might have essential roles in GC carcinogenesis.

### 2.2. miR-20a Promoted Growth of GC Cells

**Figure 2:**
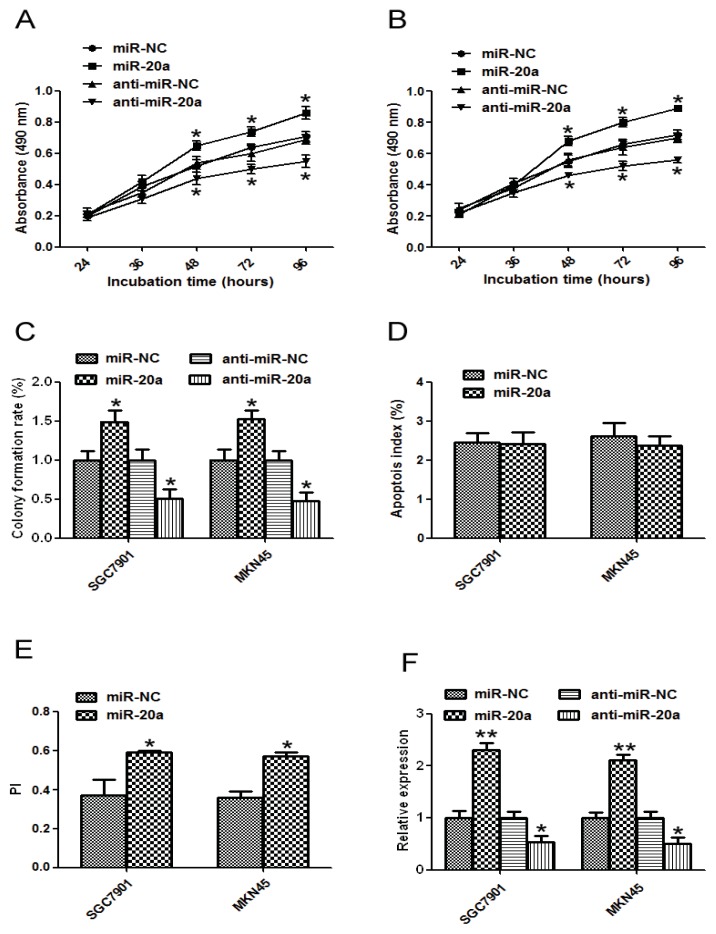
MiR-20a promoted growth of GC cell lines. SGC7901 or MKN45 cells were transfected with miR-20a precursor/inhibitor or the corresponding control, respectively. (**A**) At 24, 48, 36, 72, or 96 h after transfection, MTT assay was performed to examine SGC7901 proliferation; (**B**) MTT assay of MKN45 cells; (**C**) Representative results of colony formation assay in SGC7901 and MKN45 cells transfected with miR-20a precursor/inhibitor or the corresponding control, respectively; (**D**) The apoptosis of SGC7901 and MKN45 cells after miR-20a precursor transfection; (**E**) The proliferation index of SGC7901 and MKN45 cells transfected with miR-20a precursor; (**F**) qRT-PCR for miR-20a was performed in SGC7901 and MKN45 cells transfected with miR-20a precursor/inhibitor or the corresponding control, respectively. The data represented at least four measurements. *p < 0.05, **p < 0.01 compared with control.

As miR-20a was markedly increased in GC, it might function as a cancer promoter or suppressor. Therefore, we tested the role of miR-20a by gain- and loss- function experiments in SGC7901 and MKN45 cells. In a 3-(4,5-dimethylthiazol-2-yl)-2,5-diphenyl-tetrazolium bromide (MTT) assay, cells transfected with miR-20a precursor grew more rapidly than the control group, while miR-20a inhibitor inhibited the growth (Figure 2A,B). To further study the effect of miR-20a on the growth of GC cells, colony formation assay was performed. GC cells transfected with miR-20a precursor showed higher colony formation than cells transfected with control. However, cells transfected with miR-20a inhibitor showed lower colony formation than cells transfected with control (Figure 2C). We further used Flow cytometric analysis to determine the effect of miR-20a on apoptosis of GC cells; no significant difference was detected between miR-20a precursor and the control in these cells (Figure 2D). Results from cell cycle assay indicated that overexpression of miR-20a precursor had less cells in G0/G1 phase. Furthermore, overexpression group had more cells in S and G2M phases (Figure 2E). Proliferation index (PI) = (S + G2M)/(G0 + GS + G2M). The PI was higher in the overexpression group than that in the control group. These data suggested that miR-20a might promote SGC7901 and MKN45 cell proliferation *in vitro*. The transfection efficiency was detected by qRT-PCR (Figure 2F).

### 2.3. miR-20a Promoted Migration and Invasion of GC Cells

**Figure 3:**
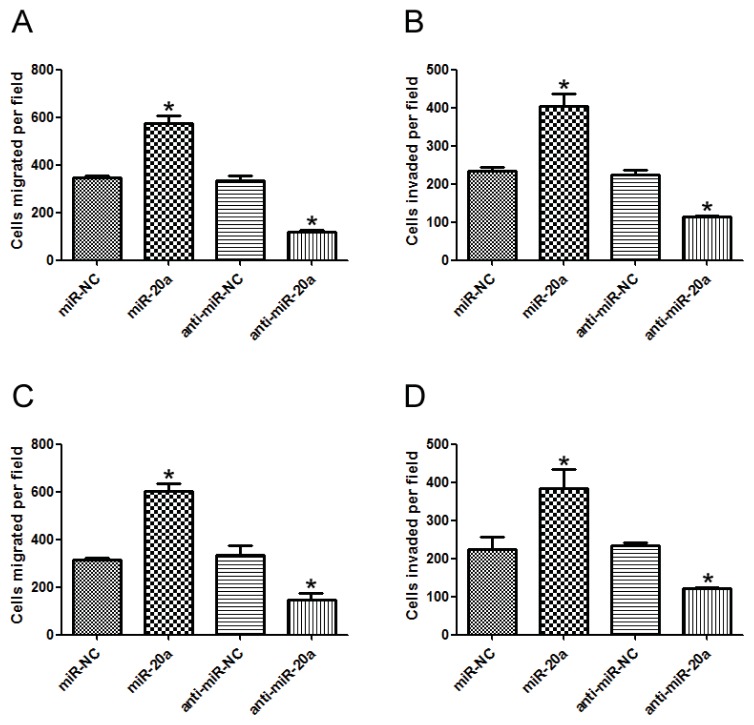
miR-20a promoted migration and invasion of GC cells. (**A**) *In vitro* migration assay of SGC7901 cells transfected with miR-20a precursor/inhibitor or the corresponding control, respectively; (**B**) *In vitro* invasion assay of SGC7901 cells transfected with miR-20a precursor/inhibitor or the corresponding control, respectively; (**C**) *In vitro* migration assay of MKN4 cells; (**D**) *In vitro* invasion assay of MKN4 cells. The data represented at least four measurements. *p < 0.05 compared with control.

To investigate the role of miR-20a in GC metastasis, miR-20a precursor/inhibitor was transfected into SGC-7901 and MKN45 cells and *in vitro* migration and invasion assays were performed. Results showed that miR-20a significantly increased the *in vitro* migration ability of GC cells, while miR-20a inhibitor remarkably decreased the *in vitro* migration ability of GC cells (Figure 3A,C). Similar results were observed in *in vitro* invasion ability of GC cells (Figure 3B,D). Collectively, these data suggested that miR-20a promoted the migration and invasion abilities of GC cells.

### 2.4. miR-20a Promoted Chemoresistance of GC Cells

**Figure 4:**
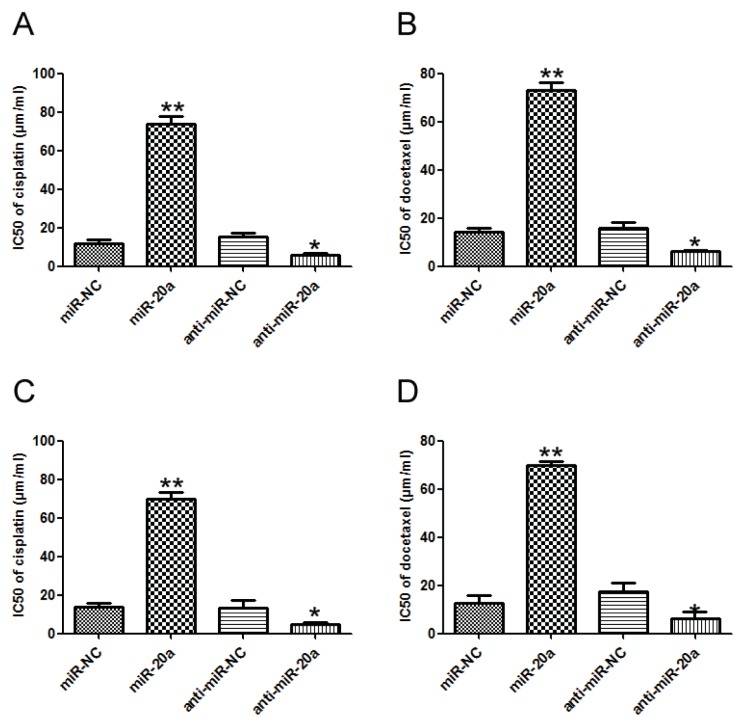
miR-20a promoted chemoresistance of GC cells. (**A**) Alteration of IC50 values (cisplatin) in SGC7901 cells transfected with miR-20a precursor/inhibitor, and the corresponding control was analyzed by MTT; (**B**) Alteration of IC50 values (docetaxel) in SGC7901 cells; (**C**) Alteration of IC50 values (cisplatin) in MKN45 cells; (**D**) Alteration of IC50 values (docetaxel) in MKN45 cells. The data represented at least four measurements. *p < 0.05, **p < 0.01 compared with control.

The effect of miR-20a on the sensitivity of GC cells to chemotherapeutic agents, cisplatin and docetaxel, was investigated. Transfection of miR-20a precursor increased the IC50 value of cisplatin, while inhibition of miR-20a decreased the IC50 value of cisplatin in SGC7901 and MKN45 cells compared with that in control group (Figure 4A,C). Similar results were obtained in docetaxel treated SGC7901 and MKN45 cells (Figure 4B,D). Our data suggested that miR-20a might promote chemoresistance of GC cells.

### 2.5. EGR2 Was a Direct Target of miR-20a

**Figure 5:**
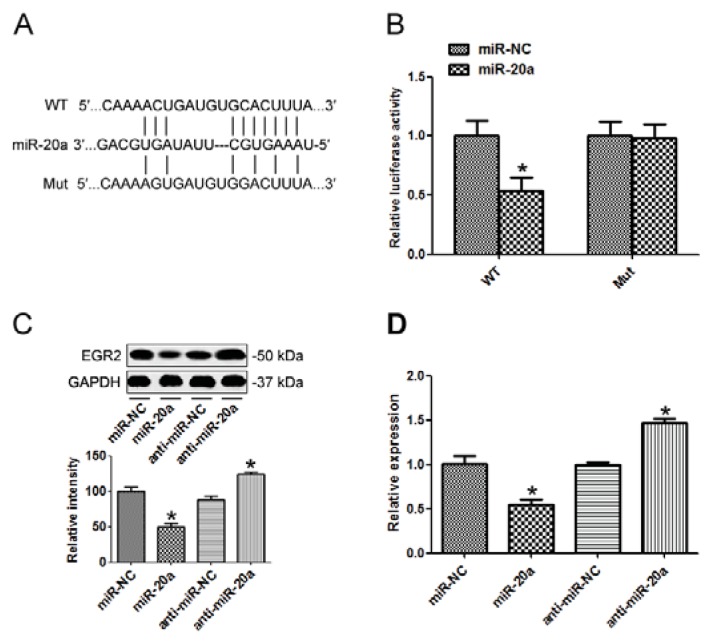
Early growth response 2 (EGR2) was a direct target of miR-20a. (**A**) Wild type of the mutated sequences of EGR2 3′ UTR (nucleotides 458-465); (**B**) SGC7901 cells were co-transfected with miR-20a precursor or negative control (miR-NC) with EGR2 3′ UTR fragment with either the miR-20a target sequence (WT), or a mutant (Mut). Luciferase activity was detected; (**C**) EGR2 protein level was detected by Western blot in cells transfected with miR-20a precursor/inhibitor or the corresponding control; (**D**) expression of EGR2 mRNA was detected by qRT-PCR in cells transfected with miR-20a precursor/inhibitor or the corresponding control. GAPDH was used as an internal control. The data represented at least four measurements. *p < 0.05 compared with control.

To investigate the downstream target of miR-20a, Targetscan 6.2 was used to screen its target. Early growth response 2 (EGR2) was predicted to be a target of miR-20a. To confirm that, we amplified the EGR2 3′ UTR containing the target sequences, or the mutants, into a luciferase reporter vector (Figure 5A). As shown in Figure 5B, miR-20a suppressed the luciferase activity of the wild type EGR2 3′ UTR (WT), while mutation of the miR-20a binding sites (Mut) blocked this suppression in SGC7901 cells. Western blot demonstrated that transfection of miR-20a precursor in SGC7901 cells inhibited EGR2 expression while miR-20a inhibitor elevated EGR2 protein level (Figure 5C). qRT-PCR showed that miR-20a precursor decreased EGR2 mRNA level, while miR-20a inhibitor elevated EGR2 mRNA level (Figure 5D), indicating that miR-20a suppressed EGR2 expression post-transcriptionally.

### 2.6. miR-20a Was inversely Correlated with EGR2 Expression

**Figure 6:**
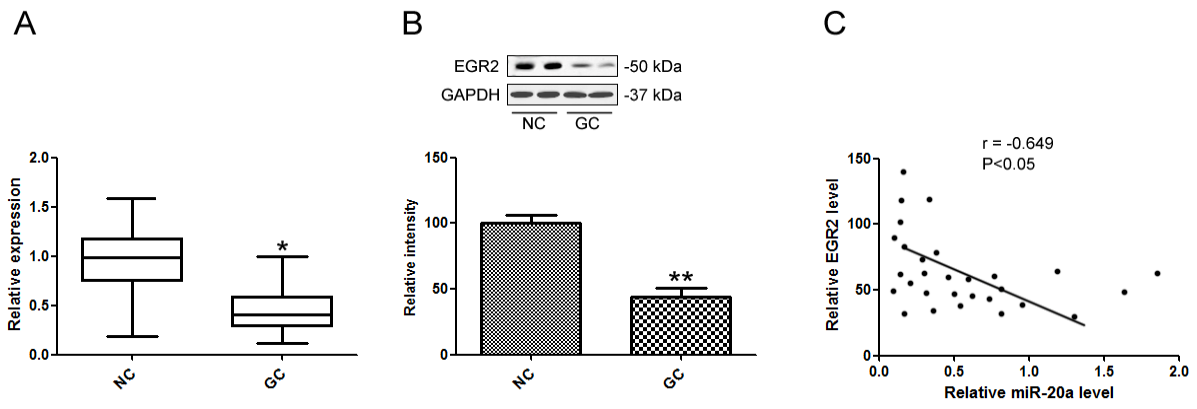
miR-20a was inversely correlated with EGR2 expression. (**A**) EGR2 mRNA levels in GC tissues were analyzed by qPCR; (**B**) EGR2 protein levels in GC tissues were analyzed by Western blot; (**C**) Correlation of miR-20a expression and EGR2 mRNA level was analyzed. The data represented triplicate measurements. *p < 0.05, **p < 0.01 compared with control.

qRT-PCR was performed to detect the mRNA levels of EGR2 in 28 GC and adjacent non-tumor normal tissue samples. EGR2 mRNA was significantly decreased in GC group (Figure 6A). Furthermore, EGR2 protein levels were also down-regulated in GC tissues compared with the non-tumor normal tissue samples (Figure 6B). Moreover, we correlated EGR2 mRNA level with miR-20a expression in the same GC tissues. EGR2 mRNA level was inversely correlated with miR-20a expression in GC tissues (Figure 6C).

### 2.7. miR-20a Promoted GC Progression by Targeting EGR2

**Figure 7:**
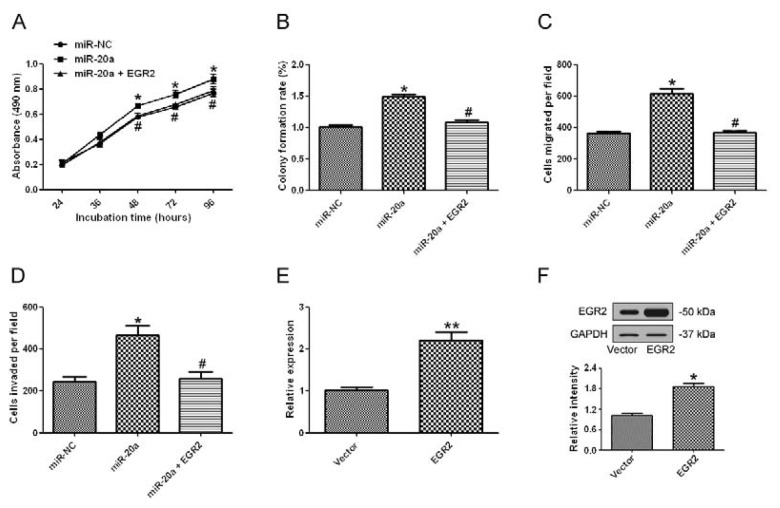
miR-20a promoted GC progression by targeting EGR2. (**A**) SGC7901 cells were co-transfected with miR-20a precursor and EGR2 overexpression plasmid or the control, at 24, 48, 36, 72, or 96 h after transfection, MTT assay was performed to examine SGC7901 proliferation; (**B**) Representative results of colony formation assay in SGC7901 cells co-transfected with miR-20a precursor and EGR2 overexpression plasmid or the control; (**C**) *In vitro* migration assay of SGC7901 cells transfected with miR-20a precursor and EGR2 overexpression plasmid or the control; (**D**) *In vitro* invasion assay of SGC7901 cells transfected with miR-20a precursor and EGR2 overexpression plasmid or the control; (**E**) qRT-PCR was used to detect the mRNA level of EGR2 in in cells transfected with EGR2 overexpression plasmid or the control; (**F**) EGR2 protein level was detected by Western blot in cells transfected with EGR2 overexpression plasmid or the control. GAPDH was used as an internal control. The data represented at least four measurements. *p < 0.05, **p < 0.01 compared with control; ^#^*p* < 0.05, compared with miR-20a precursor transfected group.

Since EGR2 was found to be a target of miR-20a, we further investigated whether overexpression of EGR2 could attenuate the oncogenic effect of miR-20a. MTT assay (Figure 7A), colony formation assay (Figure 7B), cell migration (Figure 7C) and invasion (Figure 7D) all showed that supplement of EGR2 by an EGR2 overexpression plasmid could significantly attenuate the oncogenic effect of miR-20a. The effect of EGR2 plasmid was validated by qRT-PCR (Figure 7E) and Western blot (Figure 7F). These data suggested that miR-20a promoted GC progression partially by targeting EGR2.

## 3. Discussion

Although numerous miRNAs had been identified in GC carcinogenesis, their underlying molecular mechanisms in GC development still remained poorly understood. Hence, exploring the function of miRNAs specifically involved in GC carcinogenesis would greatly help expand our understanding of GC and screening new targets for its diagnosis and therapy [18]. The aberrant expression of miR-20a in cervical cancer, colorectal cancer, GC, and prostate cancer had been found [16,19–21]. A recent study suggested that miR-20a overexpression in rheumatoid fibroblast-like synoviocytes decreased apoptosis signal-regulating kinase 1 (ASK1) activity, indicating an anti-apoptotic effect [22]. miR-20a was up-regulated in gallbladder carcinoma [23], but down-regulated in hepatocellular carcinoma [24]. The difference indicated that dysregulation of miR-20a in different cancers depended on the cellular microenvironment.

In this study, we validated that miR-20a was increased in a number of GC tissue samples and cell lines SGC7901, MKN45, and NUGC-3 cells compared to GES-1 cells. Ectopic expression of miR-20a promoted proliferation of GC cells, while suppression of miR-20a with inhibitor had the opposite effect. miR-20a was found to inhibit EGR2 expression partially by inducing mRNA decay. Similarly Wang M and colleagues reported that the levels of circulating miR-17-5p/20a might be a molecular marker for GC [20]. They found that overexpression of miR-17-5p/20a promoted GC cell cycle progression and inhibited apoptosis, whereas knockdown of miR-17-5p/20a resulted in cell cycle arrest and increased apoptosis in AGS cells and *in vivo* as well by targeting p21 and tumor protein p53-induced nuclear protein 1 (TP53INP1) [25]. In our study, overexpression of miR-20a promoted proliferation without effect on apoptosis in SGC7901 and MKN45 cell lines *in vitro*. The contradictory results might due to different cell lines, Wang M *et al.* used AGS cell line, and we used SGC7901 and MKN45 cells. Secondly, different molecules were involved in the two studies.

The MTT and colony formation assays in GC cells all suggested that forced miR-20a overexpression promoted carcinogenesis and proliferation of GC cells. Down-regulation of miR-20a expression by miR-20a inhibitor decreased these effects. Similar effects were obtained from *in vitro* migration and invasion assays. Our study suggested that miR-20a overexpression might act as an oncogene in GC.

Drug resistance remained a major obstacle for conventional chemotherapeutic agents. miRNAs had been shown to regulate drug resistance in numerous cancers [26–28]. In the present study, we found that miR-20a overexpression could significantly elevate the IC50 values of two clinical drugs, cisplatin and docetaxel, promoting the chemoresistance of GC cells.

To determine how miR-20a acted as an oncogene, we screened potential target using bioinformatics analysis, Targetscan 6.2. As a tumor suppressor, EGR2 was down-regulated in GC. Luciferase activity assay suggested direct targeting of EGR2 by miR-20a. EGR2 mRNA level was reversely correlated with miR-20a in GC patients. More importantly, supplement of EGR2 by an EGR2 overexpression plasmid could remarkably attenuate the effect of miR-20a on GC progression. EGR2 belongs to a multi-gene family encoding C2H2-type zinc-finger proteins and plays a role in the regulation of cellular proliferation [29]. Recently, some reports suggested an essential role of EGR2 in apoptosis regulation [30]. EGR2 was a target of the p53 family, and overexpression of EGR2 led to apoptosis, while down-regulation of EGR2 attenuated p53-mediated apoptosis [31]. EGR2 could be also regulated by other miRNAs. Wu Q *et al.* has reported that miR-150 could promote GC proliferation by negatively regulating EGR2 expression [32]. Our study shed new light on the regulation of EGR2.

In summary, our study validated that miR-20a was dramatically increased in GC tissues and cell lines and that ectopic expression of miR-20a promoted proliferation, migration, invasion and chemoresistance of GC cells. Moreover, down-regulation of miR-20a had the opposite effect on GC cells by targeting EGR2. Further studies for the functional and clinical implications of miR-20a and its target EGR2 might contribute to the early diagnosis and treatment of GC.

## 4. Materials and Methods

### 4.1. Cell lines and Tissue Samples

Human GC cell lines SGC7901, MKN45, NUGC-3, and human gastric mucosa cell line GES-1 were from the Chinese Academy of Sciences (Shanghai, China). Cells were grown in Dulbecco’s modified Eagle medium supplemented with 10% fetal bovine serum at 37 °C with 5% CO_2_. Twenty eight paired tissues of GC and matched normal tissues (located >5 cm away from the tumor) were collected from our department. Informed consent was obtained from each patient and this work was approved by the Ethics Committee of Third Military Medical University.

### 4.2. Plasmids and Transfection

miR-20a precursor (Catalog# HmiR0202-MR01, shown as miR-20a in figures) and inhibitor (Catalog# HmiR-AN0312-AM01, shown as anti-miR-20a) constructs were purchased from GeneCopoeia (City, MD, USA). pEGFP-N1-EGR2 plasmid was generated by using the following primers, sense 5′-CCCTCGAGATCCCAGGCTCAGTCCAACC-3′, antisense 5′-CCAAGCTTAGGTGTCCGGGTCCGAGA-3′. The amplified sequences were inserted into pEGFP-N1 within XhoI/HindIII sites. Transfection was performed using Lipofectamine 2000 reagent (Invitrogen, Carlsbad, CA, USA). Transfected cells were harvested after 24 h for migration and invasion assays and after 48 h for RNA isolation and Western blot.

### 4.3. Quantitative Real Time PCR

Total RNA was extracted using TRIzol (Invitrogen, Carlsbad, CA, USA) then reverse-transcribed into cDNA with a TaqMan MicroRNA Reverse-Transcription Kit (Applied Biosystems, Foster City, CA, USA). PCR reactions were performed using the ABI Stepone plus Detection System (Applied Biosystems, Foster City, CA, USA). The relative expression of miRNA was normalized with U6. Samples were compared by using the relative CT method. The fold change was determined relative to a control after normalizing to a housekeeping gene by using 2^−ΔΔCT^, where ACT is (gene of interest CT) minus (GAPDH CT), and ΔΔCT is (ΔCT cancer) minus (ΔCT control). The relative expression of EGR2 was normalized with GAPDH. All experiments were carried out at least in triplicate.

### 4.4. *In Vitro* Cell Proliferation Assay

Forty eight hours after transfection, cells were seeded into 96-well plates at 6 × 10^3^ cells/well. The MTT assay was used to measure cell viability. Optical densities at 490 nm were measured.

### 4.5. Colony Formation Assay

Cells transfected with miR-20a precursor/inhibitor or the corresponding control were seeded in a 10 cm dish and maintained in complete culture medium. After 21 days, SGC7901 cells were fixed with methanol and then stained with 0.1% crystal violet. Colonies were manually counted.

### 4.6. *In Vitro* Migration and Invasion Assay

*In vitro* migration assay was performed using 8 μm pore size Transwell plates (Millipore, Billerica, MA, USA). After transfection, the cells (1 × 10^5^ cells/100 μL serum-free medium) were added to the upper chamber. RPMI 1640 containing 10% FCS was added to the bottom chamber as a chemoattractant. After 24 h, cells on the upper surface were removed, while cells attached on the bottom were fixed and stained with 0.1% crystal violet. The images of invaded cells were counted with a photomicroscope (Olympus, Tokyo, Japan). For the *in vitro* invasion assay, the plates were coated with matrigel (BD Biosciences, San Jose, CA, USA) diluted in serum-free medium and performed the same as migration assay.

### 4.7. Luciferase Reporter Assay

The 3′ UTR of human EGR2 was PCR-amplified and cloned into psiCHECK-2 vector. These constructs (1 μg) were co-transfected with 1 μg of control or miR-20a precursor into SGC7901 cell. Luciferase activity was assayed 48 h after transfection by using the Dual-luciferase activity assay system (Promega, Madison, WI, USA). All the experiments were performed at least in four times.

### 4.8. Western Blot

Cells were harvested 48 h after transfection, and then proteins were extracted and separated by 10% SDS-PAGE. Gels were transferred to nitrocellulose filter membrane and probed with anti-EGR2 or anti-GAPDH. Horseradish peroxidase-conjugated secondary antibodies were applied and detected by enhanced chemiluminescence (Thermo Scientific, Rockford, IL, USA).

### 4.9. Flow Cytometric Analysis of Apoptosis

Cells were harvested at the above indicated time points, at least 5 × 10^5^ cells were recovered by centrifugation for evaluation of apoptotic cells with the use of double staining with annexin V-fluoresein isothiocyanate (annexin V-FITC) and propidium iodide (PI) (BioVision, St. Pete Beach, FL, USA) according to the manufacturer’s instructions, followed by flow cytometric analysis with the use of Cell Quest software (Version 5.1, Becton, Rutherford, NJ, USA).

### 4.10. Cell Cycle Analysis by Flow Cytometry

Cells were harvested 48 h after transfection and fixed in 75% ethanol. Then, cells were treated with RNase A and PI (50 μg/mL) and incubated for 30 min. Cell cycle analysis was performed by Flow cytometry.

### 4.11. Statistical Analysis

All experiments were repeated independently at least in triplicate, and the results were expressed as the mean ± SD. The results were assessed by a one-way ANOVA, or Student *t* test. A value of *p* < 0.05 was accepted to indicate statistical significance.

## 5. Conclusions

Our work demonstrated that miR-20a was involved in the carcinogenesis of GC through modulation of the EGR2 signaling pathway.

## Figures and Tables

**Table 1:** Correlation of the expression of miR-20a with clinicopathologic features.

Clinicopathologic features	No.	Relative expression of miR-20a a	*p*-value b
Gender			0.727
Male	18	0.72 (0.51–1.33)	
Female	10	0.70 (0.52–1.02)	
Site of tumor			0.810
Upper stomach	8	0.68 (0.52–1.00)	
Middle stomach	6	0.70 (0.51–1.22)	
Lower stomach	14	0.71 (0.51–1.32)	
Differentiation			0.655
Poor	12	0.73 (0.59–1.33)	
Moderate	16	0.72 (0.52–1.12)	
Metastasis			0.003
N0	4	0.55 (0.51–0.61)	
N1	5	0.62 (0.51–0.64)	
N2	9	0.74 (0.52–1.09)	
N3	9	0.92 (0.51–1.33)	

a Median of relative expression;

b Mann-Whitney U test between two groups and Kruskall-Wallis test for three groups.
